# Technoeconomic assumptions adopted for the development of a long-term electricity supply model for Cyprus

**DOI:** 10.1016/j.dib.2017.08.019

**Published:** 2017-09-05

**Authors:** Constantinos Taliotis, Emanuele Taibi, Mark Howells, Holger Rogner, Morgan Bazilian, Manuel Welsch

**Affiliations:** a KTH – Royal Institute of Technology, division of Energy Systems Analysis, Office K514, Brinellvägen 68, 100 44 Stockholm, Sweden; b International Renewable Energy Agency (IRENA), Robert Schuman Platz 3, 53111 Bonn, Germany

**Keywords:** Renewable energy, Cost-optimization, Cyprus, Scenarios, Energy policy, MESSAGE

## Abstract

The generation mix of Cyprus has been dominated by oil products for decades. In order to conform with European Union and international legislation, a transformation of the supply system is called for. Energy system models can facilitate energy planning into the future, but a large volume of data is required to populate such models. The present data article provides information on key modelling assumptions and input data adopted with the aim of representing the electricity supply system of Cyprus in a separate research article. Data in regards to renewable energy technoeconomic characteristics and investment cost projections, fossil fuel price projections, storage technology characteristics and system operation assumptions are described in this article.

**Specifications Table**

**Table:** 

Subject area	*Engineering*
More specific subject area	*Energy Technology*
Type of data	*Description of main assumptions, tables and figures with model input data*
How data was acquired	*Literature survey (reports from international organizations and journal articles)*
Data format	*Descriptive*
Experimental factors	*Not applicable*
Experimental features	*Not applicable*
Data source location	*Not applicable*
Data accessibility	*Data is available within this article*

**Value of the data**

• The data provided are required to understand the conditions under which RET technologies are deemed cost-competitive in Cyprus.
• Providing detailed data is important in keeping the process of energy policy-making transparent. This facilitates in building consensus between stakeholders.
• Local universities can take up the developed model and adjust data accordingly to extend the analysis.

## 1 Data

This data article relates to the analysis conducted in a long-term cost-optimization model focused on the electricity supply system of Cyprus [1]. Data and key assumptions adopted in the analysis are described below in three sections.

### 1.1 Main modelling assumptions

#### 1.1.1 Electricity supply system

Beyond the existing, committed and planned power plant projects mentioned above, future investments in fossil-fueled and renewable energy technologies are allowed to occur so as to expand the generating capacity of the system, if required, to meet growing demand. Technology options available and assumptions about their parameters can be found in Section 1.2.

In regards to renewable energy technologies, any installations beyond the existing and committed levels (a total of 50 MW CSP with storage, 15 MW distribution-connected PV under net-metering yearly up to 2020, a total of 175 MW wind by 2018) are simply part of the model's minimum cost pathway to satisfy electricity demand. Generic cost assumptions have been adopted in the analysis, whenever specific data for Cyprus were not available. However, the data is aligned with the situation in Cyprus. For instance, the IEA projects investment costs for rooftop PV in 2014 at 2900 EUR/kW [2], while at the moment installations in Cyprus cost 1400–2000 EUR/kW. Previous IRENA assessments report values that are within this range [3], [4], so IRENA values are adopted for PV (investment cost of €1665/kW for 2013).

**Fig. 1:**
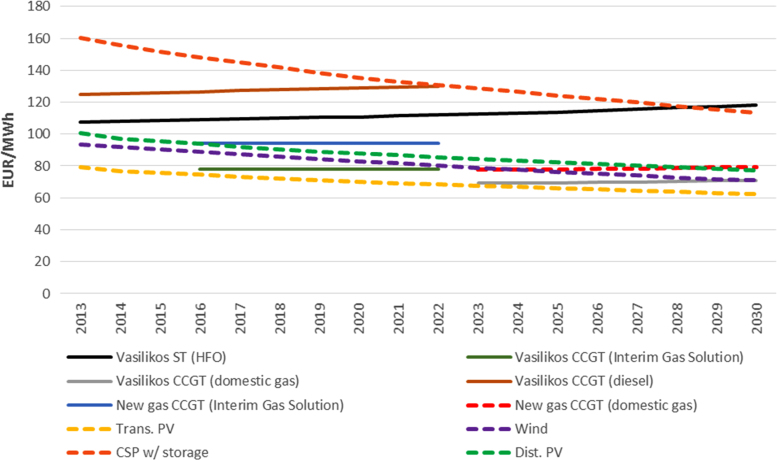
Generation cost comparison: short-run marginal cost for the Vasilikos Power Plant (using different fuels) and long-run marginal cost for new gas CCGT, solar PV, CSP and wind.

With the assumptions taken on technology costs and performance, as well as fuel prices, solar PV connected at the transmission level appears to be the cheapest electricity generation technology for Cyprus at the moment (Fig. 1). Only the Vasilikos combined cycle gas turbines might have a comparable generation cost in the future, depending on the fuel price at which natural gas will be purchased either from external or future domestic suppliers.

Additional to power generating technologies, storage options are considered. The technologies considered in the analysis are pumped-hydro storage [5], flow batteries for transmission-connected storage and Li-ion batteries for storage at the distribution level [6]. Detailed assumptions regarding storage options can be found in Section 1.3.

#### 1.1.2 Fuel assumptions

Currently, heavy fuel oil, diesel and small quantities of biomass are the fuels used for power generation in Cyprus. Nonetheless, once natural gas becomes available, it will become the main fuel for power generation, either through the Interim Gas Solution, subject to its economic feasibility, or through domestic production. Recorded fuel prices from the island or from Europe are taken and related to international crude oil prices [7]. Hence, available price projections for crude oil [8] are used to establish price projections for heavy fuel oil, low-S fuel oil and diesel (Table 1).

**Table 1:** Projected fuel prices used in the model.

		**2013**	**2015**	**2020**	**2025**	**2030**	**2035**
**Oil**	**USD/barrel**	110	111	113	116	121	128
	**EUR/Mbtu**	14.1	14.7	15.1	15.7	16.6	14.1
**Heavy Fuel Oil**	**EUR/Mbtu**	11.7	11.8	12.1	12.4	12.9	13.6
**Diesel**	**EUR/Mbtu**	16.8	16.9	17.3	17.8	18.5	19.6
**Low-S Fuel Oil**	**EUR/Mbtu**	14.5	16.1	16.5	16.9	17.6	18.6
**Natural Gas (Int. Market Price)**^a^	**EUR/Mbtu**	8.8	8.9	9.0	9.0	9.3	9.6

a Market price is aligned with the projected European market price and is the assumed price for domestic use in the power sector [8].

In order to calculate a reasonable price of gas for the Interim Gas Solution, the assumption as indicated by MECIT is to use a price at which generation cost would be comparable to that of the cheapest renewable energy technology, based on the current price assumptions. As such, the price of imported natural gas (interim gas solution) is set at a level so as the variable cost of generation from the most efficient gas-firing power plants (i.e. existing Vasilikos CCGT) matches that of transmission connected solar PV.^1^ It is assumed that domestic natural gas will be purchased at market price, and thus projections at the European level [8] have been used. Nonetheless, a sensitivity analysis is conducted to investigate the potential effect of a range of gas prices on the generation mix. An additional important assumption was taken in regards to the interim gas solution relating to the volume of gas imports. A limit is imposed on the maximum generation from renewables during the years where imports occur, in order to simulate a take-or-pay agreement, which would force purchase of a minimum volume of gas.

#### 1.1.3 Concerns regarding system reliability

System reliability will change with a higher share of variable renewable energy; especially with a lack of an interconnection to external grid networks. However, if the interconnector with Israel and Greece is developed, Cyprus could gain access to the reserves offered by these countries, provided market design will allow for efficient cross-border trading of electricity and ancillary services. Furthermore, curtailment of electricity from variable renewables can be tackled through exports at a low price. This sub-section details the assumptions taken in the analysis regarding system reliability and reserve requirements.

##### 1.1.3.1 System operational reserve requirements

In order to minimize the risk of power shortages caused by rapid changes in power output from variable renewable energy technologies or potential outages of thermal plants, operational reserves are required by the system. A rather conservative approach is taken regarding minimum requirements of fast-response (spinning) reserves. Spinning reserve can be described as online units that can be ramped up or down within seconds or minutes to limit sudden shifts in the frequency of the grid, for example due to outages [9]. Based on information provided by the Transmission System Operator in Cyprus spinning reserve requirements are the following:

• At all times at least 60 MW of spinning reserve needs to be available beyond the level generated to cover demand for electricity.
• An additional reserve corresponding to 50% of the predicted wind generation should be available.
• In addition to the above, fast-response reserve is needed to account for 10% of the predicted solar PV and CSP generation without storage.

In this study, it is assumed that all existing and future centralized thermal plants are allowed to contribute to the above reserves, assuming that all remaining spinning capacity is fast-response-reserves. It is important to note that the proposed interconnector with Greece and Israel, along with associated reserves in the respective countries, may partly contribute to these reserves, but this decision is yet to be made. As such, the aforementioned reserve requirements are kept the same in all scenarios.

##### 1.1.3.2 System capacity reserve requirements

In terms of capacity reserve margin, no such constraint has been imposed in the model. It is assumed that the renewable energy technologies, the potential interconnector, and the storage facilities cover the security of the system with respect to available capacity of power generation sources to cover the needs of the system. These assumptions need to be re-examined in future analyses, as the contribution to “firm” capacity of each renewable energy technology, storage facilities or the interconnector is evaluated. It should be noted that by extending the lifetime of the existing installations at Vasilikos, Dhekelia and Moni, such a capacity reserve can be maintained well beyond 2030.

##### 1.1.3.3 Limitations related to the penetration of solar PV and wind

It is beyond the scope of the present study to examine in detail grid integration issues. Therefore, in order to address a potential unrealistic deployment of large shares of variable renewable technologies in the absence of an interconnector, limitations are placed on the amount of solar PV and wind that can enter the system without associated storage:

• The maximum capacity of solar PV without storage is set at 400 MW by 2018.
• The corresponding limit for wind is set at 200 MW by 2018.

These limits are based on preliminary results from a separate study conducted by the Cypriot authorities and these capacities represent respectively 40% and 20% of the peak demand for 2018 (CERA, 2014). In the present analysis, these limits are allowed to increase after 2018 following demand growth and maintaining the same share on peak electricity demand. In this sense, the capacity limits reach 550 and 275 MW by 2030 in the Energy Efficiency scenario, and 468 and 234 MW in the Extra Efficiency scenario for solar PV and wind respectively. If these thresholds are reached by the model, no new solar PV without storage or wind can enter the generation mix. Instead, the system can either

• invest in decentralized storage options of 1 kWp for each 1 kWp of distribution-connected solar PV; or
• invest in transmission connected storage (i.e. pumped hydro storage or flow batteries). For instance, in case 130 MW pumped hydro storage is deployed, an additional 130 MW of solar PV or wind can be installed.

These are simplified assumptions based on the best available information at the time of the study. A detailed grid model is under development by the Joint Research Centre of the European Commission. Based on the findings of this grid study, an update of the model can be developed, and a suite of grid integration measures that can be adopted will be assessed. Specific issues related to grid codes and market design will also influence the amount to variable renewables that can be integrated.

### 1.2 Detailed power plant assumptions for future projects

See Table 2, Table 3.

**Table 2:** Plant cost and performance parameters for future projects.

**Technology type**	**Input fuel**	**Efficiency (%)**	**Variable operation and maintenance cost (EUR /MWh)**	**Fixed operation and maintenance cost (EUR/kW)**	**2013 Inv cost (EUR /kW)**	**Capacity factor (%)**	**First year**	**Construction time (years)**	**Plant life (years)**
**Combined Cycle**	Gas	47.50		33.1	828	86.02	2019	3	30
**Gas Turbine**	Diesel/Gas	44.00		27.1	677	82.80	2019	2	30
**Steam Turbine**	HFO or Low-S FO	38.46		27.1	1016	80.10	2019	2	30
**Wind**			14.3		1310	16.00	2015	1	25
**Biomass**		32.00		75.3	2800	48.50	2015	2	25
**Solar PV utility**			15.1		1332	18.50	2015	1	25
**Solar PV rooftop**			11.3		1665	18.50	2014	<1	20
**CSP w/ storage**			21.8		6200	39.25	2017	2	25

**Table 3:** RE technology investment cost projections.

	**EUR/kW**								
	**2013**	**2014**	**2015**	**2016**	**2017**	**2018**	**2019**	**2020**	**2021**
**Wind**	1417	1389	1361	1334	1307	1281	1255	1230	1205
**Biogas-biomass**	2800	2748	2695	2643	2590	2537	2485	2432	2406
**Solar PV utility**	1332	1279	1253	1228	1203	1179	1156	1138	1121
**PV rooftop**	1665	1598	1566	1535	1504	1474	1445	1423	1402
**CSP w/ storage**	6200	6009	5818	5679	5541	5403	5264	5126	5032
	**2022**	**2023**	**2024**	**2025**	**2026**	**2027**	**2028**	**2029**	**2030**
**Wind**	1181	1158	1135	1112	1090	1068	1046	1026	1020
**Biogas-biomass**	2380	2353	2327	2301	2275	2248	2222	2196	2169
**Solar PV utility**	1105	1088	1072	1056	1040	1024	1009	994	984
**PV rooftop**	1381	1360	1340	1320	1300	1280	1261	1242	1230
**CSP w/ storage**	4938	4844	4751	4657	4563	4469	4376	4282	4188

### 1.3 Detailed electrical storage assumptions

See Table 4, Table 5.

**Table 4:** Pumped-hydro storage specifications [5].

**Location of facility**	**Kourris Dam**
**Year of Operation**	2021
**Nominal Capacity (MW)**	130
**Overall efficiency**	77%
**Full load operation for electricity production**	88 h
**Capital cost (EUR/kW)**	1185
**O&M cost (EUR/kW-yr)**	11

**Table 5:** Battery options for electrical storage [6].

	**Flow batteries**	**Li-ion batteries**
**Level**	Centralized	Decentralized
**First Year of Operation**	2016	2016
**Capital cost EUR/kW**	828	527
**Capital cost EUR/kWh**_**cap**_	433	753
**Fixed OM cost EUR/kW-yr**	22.6	18.8
**Efficiency**	77.5%	90%
**Lifetime (yrs)**	10	12.5
**Lifetime (charge cycles)**	1500–15,000	2000–3000

## 2 Experimental design, materials and methods

A literature survey was carried out on reports from international organizations, journal articles and sources from local universities and authorities in Cyprus. The data was compiled, presented and discussed with local stakeholders so as to reach consensus on the main data and assumptions to be used in the analysis.

## Footnotes

1 In the case of PV costs considered include investment, operation and maintenance costs, while the lifetime, capacity factor and associated discount rate are also taken into account.
