# Supplementary material for: Technoeconomic assumptions adopted for the development of a long-term electricity supply model for Cyprus
Source: Data Brief. 2017 Sep 5;14:730–7. doi: 10.1016/j.dib.2017.08.019 (PMC5596323; doi:10.1016/j.dib.2017.08.019)
Supplement: Supplementary file 1 — Supplementary material [file mmc1.pdf]

Conflict of Interest

We wish to confirm that there are no known conflicts of interest associated with this publication and the financial support for this work received for this work has not influenced its outcome.

We confirm that the manuscript has been read and approved by all named authors and that there are no other persons who satisfied the criteria for authorship but are not listed. We further confirm that the order of authors listed in the manuscript has been approved by all of us.

We confirm that we have given due consideration to the protection of intellectual property associated with this work and that there are no impediments to publication, including the timing of publication, with respect to intellectual property. In so doing we confirm that we have followed the regulations of our institutions concerning intellectual property.

On behalf of the authoring team,

Constantinos Taliotis
